# Genome Assessment of Carbapenem- and Colistin-Resistant *Escherichia coli* from Patients in a Sentinel Hospital in China

**DOI:** 10.3390/cells11213480

**Published:** 2022-11-03

**Authors:** Jiangang Ma, Biao Tang, Jiahui Lin, Abdelaziz Ed-Dra, Hui Lin, Jing Wu, Yuzhi Dong, Hua Yang, Min Yue

**Affiliations:** 1 State Key Laboratory for Managing Biotic and Chemical Threats to the Quality and Safety of Agro-Products & Institute of Agro-Product Safety and Nutrition, Zhejiang Academy of Agricultural Sciences, Hangzhou 310021, China; 2 Hainan Institute of Zhejiang University, Sanya 572025, China; 3 Institute of Preventive Veterinary Sciences and Department of Veterinary Medicine, Zhejiang University College of Animal Sciences, Hangzhou 310058, China; 4 Zhejiang Provincial Key Laboratory of Preventive Veterinary Medicine, Hangzhou 310058, China; 5 State Key Laboratory for Diagnosis and Treatment of Infectious Diseases, National Clinical Research Center for Infectious Diseases, National Medical Center for Infectious Diseases, The First Affiliated Hospital, College of Medicine, Zhejiang University, Hangzhou 310058, China

**Keywords:** *bla*_NDM_, *Escherichia coli*, inpatient, *mcr-1*, *astA*

## Abstract

Antimicrobial-resistant (AMR) pathogens are a significant threat to public health worldwide. However, the primary carrier of AMR genes, particularly against last-resort antibiotics, is still only partially studied in Chinese hospitals. In a sentinel hospital in China, we collected 157 *E. coli* strains from patients between January and July 2021. One *bla*_NDM-1_-, nine *bla*_NDM-5_-, and one *mcr-1*-positive *E. coli* recovered from inpatients were identified as resistant to meropenem and colistin. There are 37 virulence genes discovered in the 11 strains, including *astA* in strain EC21Z-147 (O128: H4), which belongs to the enteroaggregative *E. coli* (EAEC). The *bla*_NDM_ gene is distributed into distinct ST types, including ST48, ST616, ST410, ST711, and ST2003, while the *mcr-1* gene was identified in ST117. The conjugative plasmids IncX3, IncI1-I, and IncI2 mediated the *bla*_NDM-5_ and *mcr-1* genes detected among inpatients. Notably, the youngest age at which *mcr-1*-positive *E. coli* has been reported was at one day old, in a child in which the strain is closely related to strains with animal origins. Hospitals are major environments for the spread and dissemination of critical virulence and AMR genes, which requires active monitoring systems at the genome level to surveil the spread of virulence and AMR.

## 1. Introduction

Antimicrobial resistance (AMR) has become a global public health concern with the spread of multidrug resistance (MDR) bacteria worldwide. Carbapenems are the most critical antimicrobials for treating clinical Gram-negative bacterial infections. The carbapenem-resistant *Enterobacteriaceae* (CRE), defined as a superbug that carried the *bla*_NDM-1_ gene, was identified in 2009 [1]. *bla*_NDM_ is a metallo-β-lactamase (MBL) type gene that could hydrolyze most β-lactam antimicrobials [2]. Hence, they are considered last-resort antimicrobials against serious infections caused by CRE [3]. Moreover, colistin is considered among the last resort for treating MDR *Enterobacteriaceae*; however, its extensive use in veterinary practice has led to the development of resistance in a wide range of pathogens recovered from animals, food, the environment, and human samples [4,5,6,7]. On the other hand, tigecycline is a last-resort antibiotic used to treat severe infections caused by extensively drug-resistant pathogenic bacteria [8]. Hence, resistance to these last-resort antimicrobial drugs threatens the healthcare systems.

Resistance to these antimicrobials is encoded by the antimicrobial resistance gene (ARG) carried on transferable plasmids, further complicating the situation and accelerating their dissemination worldwide. So far, thirty-one variants of *bla*_NDM_, ten variants of *mcr*, and dozens of *tet*(X) variants encoding resistance to carbapenem, colistin, and tigecycline have been identified in various bacterial species globally [9,10,11,12,13]. These ARGs have been reported in several incompatibility groups of plasmids, including IncF, IncFII, IncN, IncHI, IncX, and others [14,15].

Hospital-associated and community-acquired infections are important routes of transmission of ARGs [16]. *Escherichia coli* is considered one of the most common bacterial species responsible for the horizontal transfer of ARGs [17]. Hence, to estimate the trend and transfer mechanisms of carbapenems, polymyxins, and tigecycline resistance genes in patients, here, we analyzed the prevalence of meropenem-, colistin- and tigecycline-resistant *E. coli* strains for inpatients and compared them with outpatients in the same period in a sentinel hospital in Zhejiang province, China. The molecular characteristics and the capability of horizontally transferring the *bla*_NDM_ and *mcr-1* genes were also assessed.

## 2. Materials and Methods

### 2.1. Bacteria Isolation and Identification

A total of 157 *E. coli* strains from 139 patients were retrospectively collected from outpatients (*n* = 69) and inpatients (*n* = 88) between January and July 2021 at a sentinel hospital in Zhejiang province, China. The isolated strains were confirmed with MALDI-TOF Mass Spectrometry. These strains were recovered from blood (*n* = 65), urine (*n* = 48), stool (*n* = 14), bile (*n* = 23), and lung (*n* = 7) samples. The patients’ ages were between 1 day and 100 years old.

### 2.2. Antimicrobial Susceptibility Testing

The *E. coli* isolates were first tested against meropenem (2 mg/L), colistin (2 mg/L), and tigecycline (2 mg/L) to select the resistant strains by using the diffusion method on Mueller–Hinton agar (MHA). The genes encoding resistance to carbapenem (*bla*_NDM_) and colistin (*mcr-1*) were then amplified using the primers previously described [18,19]. Thereafter, the minimum inhibitory concentrations (MICs) were determined for 11 meropenem and/or colistin-resistant strains from 14 antimicrobial agents, including ampicillin (AMP), amoxicillin/clavulanate (A/C), gentamicin (GEN), spectinomycin (SPT), florfenicol (FFC), sulfisoxazole (SF), trimethoprim/sulfamethoxazole (SXT), ceftiofur (CEF), ceftazidime (CAZ), enrofloxacin (ENR), ofloxacin (OFL), tetracycline (TET), colistin (COL), and meropenem (MEM) using the broth microdilution method according to the guidelines of the Clinical and Laboratory Standards Institute (CLSI) [20].

### 2.3. Whole-Genome Sequencing and Annotation

The genomic DNA of the 11 colistin- or meropenem-resistant *E. coli* strains was extracted using a DNA Extraction Kit (Genray, Shanghai, China). Whole-genome sequencing was performed by the Oxford Nanopore GridION platform for long sequencing reads and Illumina HiSeq-PE150 for High-Throughput Sequencing reads. The genome sequences were hybrid assembled using Unicycler v0.4.4. The sequences were automatically annotated by the RAST [21]. The ARGs were identified using the ResFinder 4.1 (>90% identity and >80% coverage) (https://cge.food.dtu.dk/services/ResFinder/) (accessed on 15 June 2022). The virulence genes were identified using the VirulenceFinder 2.0 (>90% identity and >80% coverage) (https://cge.food.dtu.dk/services/VirulenceFinder/) (accessed on 15 June 2022), and the incompatibility (Inc) group of plasmids were identified using the PlasmidFinder 2.1 (>95% identity and >80% coverage) (https://cge.food.dtu.dk/services/PlasmidFinder/) (accessed on 15 June 2022), as reported in previous studies [22,23,24,25,26]. Insertion sequences were identified by ISFinder [27].

### 2.4. S1-Pulsed-Field Gel Electrophoresis (PFGE) and Southern Blot

As described in a previous study, the 11 *E. coli* strains were subjected to S1-PFGE to identify the number and size of plasmids [28,29]. The overnight cultures were washed with PBS buffer and then embedded in agarose plugs. The plugs were digested with proteinase K at 37 °C, shaking at 120 rpm for 2 h, and were restricted with S1 nuclease. *Salmonella* H9812 was restricted with *Xba*I, which was used as the size marker. A Southern blot was used to confirm the positioning of the *mcr-1* or *bla*_NDM_ genes on the plasmids. The DNA fragments were transferred to a positively charged nylon membrane (Millipore, USA) by wet transfer and then hybridized according to the protocol as the DIG-High Prime DNA Labeling and Detection Starter Kit I (Roche, Germany).

### 2.5. Conjugation Test

Colistin- or meropenem-resistant *E. coli* containing the *mcr-1*, *bla*_NDM-1_, or *bla*_NDM-5_ genes was used as the donor strain, and *E. coli* J53 (sodium azide-resistant) was used as the recipient. Fresh cultures of the donor and recipient strains were adjusted in concentration and co-incubated on the LB agar plate at 37 °C overnight. The mixed cultures were collected and diluted with PBS. Then, the mixture was inoculated onto MHA plates containing 2 µg/mL of colistin with NaN_3_ (100 µg/mL) or 2 µg/mL meropenem with NaN_3_ (100 µg/mL) at 37 °C overnight. The number of transconjugants and recipients was recorded, which was used to calculate the transfer frequency as described previously [18].

## 3. Results

### 3.1. Bacterial Strains

A total of 157 *E. coli* strains were collected from outpatients and inpatients between January and July 2021 in Zhejiang province, China. Ten (6.37%, 10/157) meropenem-resistant strains were isolated from the stool and lungs of inpatients (Table 1). Among them, four strains (EC21Z-078, EC21Z-083, EC21Z-097, and EC21Z-101) were recovered from the same patient (No. 01262726) with diffuse large B-cell lymphoma at different times, and two strains (EC21Z-144 and EC21Z-151) were isolated from another patient (No. 01273986) with non-Hodgkin’s lymphoma. A total of nine (5.73%) *bla*_NDM-5_- and one (0.64%) *bla*_NDM-1_-harboring strains were detected, while only one (0.64%) colistin-resistant strain carrying the *mcr-1* gene was isolated from the lung of a one-day-old inpatient child (Table 1). Importantly, screening of the studied isolates (*n* = 157) toward tigecycline showed that all the tested strains were susceptible. It is important to note that all 11 resistant strains were isolated from inpatients (12.5%, 11/88) instead of outpatients (0%, 0/69).

### 3.2. Antimicrobial Resistance Profile

The susceptibility testing results showed that all 11 strains (100%) were resistant to ampicillin, sulfisoxazole, and ceftiofur, and most of them exhibited resistance against amoxicillin/clavulanate (90.9%), ceftazidime (90.9%), spectinomycin (81.8%), florfenicol (81.8%), trimethoprim/sulfamethoxazole (81.8%), tetracycline (72.7%), enrofloxacin (72.7%), and ofloxacin (72.7%). However, only three (27.3%) strains were resistant to gentamicin (Table 2). Importantly, all 11 examined strains showed an MDR. It is noted that despite the strains EC21Z-078, EC21Z-083, EC21Z-097, and EC21Z-101 being isolated from one patient at a different time, they did not present identical antimicrobial resistance profiles.

### 3.3. Characterization of the bla_NDM-1_, bla_NDM-5_, and mcr-1 Genes

**Figure 1:**
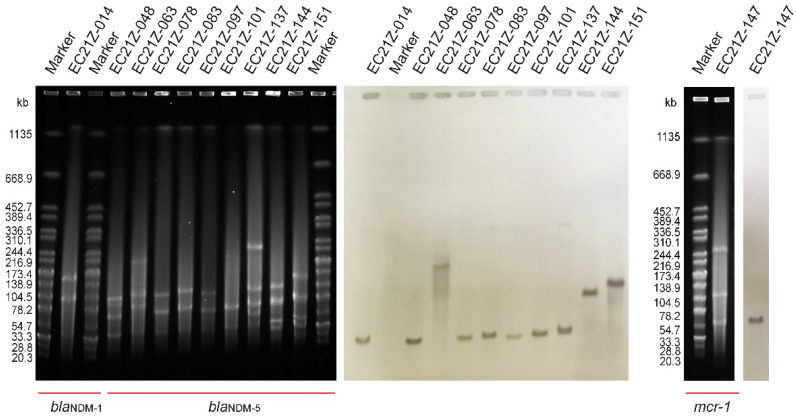
S1-PFGE and Southern blot of *bla*_NDM-1_-, *bla*_NDM-5_-, and *mcr-1*-bearing *E. coli* strains (*n* = 11).

The results of S1-PFGE showed that all 11 strains contain more than two plasmids. The sizes of the plasmids were concentrated between 20.5 kb and 244.4 kb. One plasmid (~90 kb) was missed in the strain EC21Z-101 compared to the first three isolates from the same patient. The Southern blot analysis demonstrated that all *bla*_NDM-1_, *bla*_NDM-5_ and *mcr-1* genes were located on the plasmids (Figure 1). Among them, the *bla*_NDM-1_, *mcr-1*, and most *bla*_NDM-5_ genes were found on similar size (~50 kb) plasmids that may have similar Inc types. Moreover, the strain EC21Z-063 contained a large plasmid (~200 kb) carrying *bla*_NDM-5_. The strain EC21Z-151 had a slightly larger plasmid than strain EC21Z-144, both isolated from a patient.

**Figure 2:**
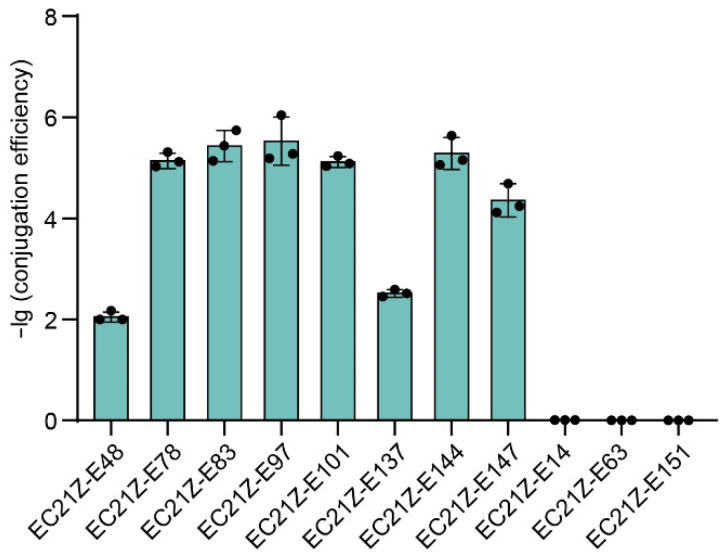
The conjugation transfer frequency of 11 *bla*_NDM_- or *mcr*-positive *E. coli* strains.

Conjugation experiments showed that the *bla*_NDM-1_ gene carried on the plasmid in strain EC21Z-014 could not be transferred to the recipient *E. coli* J53. Seven of nine plasmids carrying *bla*_NDM-5_ (strains EC21Z-048, EC21Z-078, EC21Z-083, EC21Z-097, EC21Z-101, EC21Z-137, and EC21Z-144) were successfully transferred to recipient *E. coli* J53 with a conjugation frequency in the range from 1 × 10^−2^ to 5 × 10^−6^ per recipient strain. Interestingly, the conjugation ability was different for different strains, EC21Z-144 and EC21Z-151, isolated from one patient. The plasmid carrying the *mcr-1* gene from strain EC21Z-147 was transferred to the recipient at 4.79 × 10^−5^ conjugation frequency (Figure 2).

### 3.4. Genomic Characteristics Analysis

**Figure 3:**
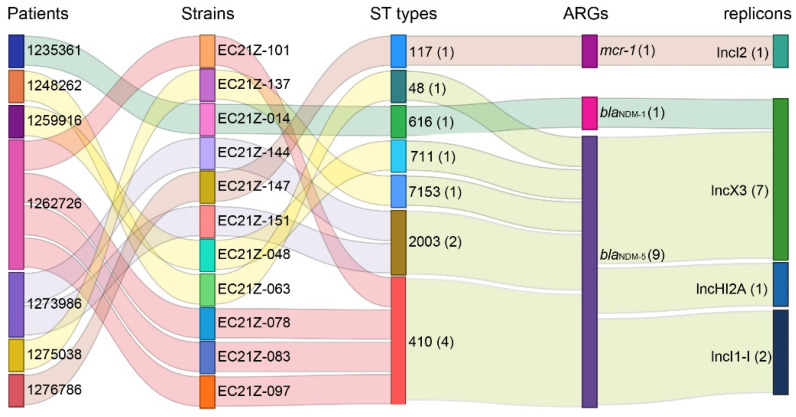
The relationship of the resistant strains from the patient with the ST types, *bla*_NDM-1_, *bla*_NDM-5_, and *mcr-1* genes and their bearing plasmids. The strain numbers are marked behind the information with parentheses.

The genomes of all *bla*_NDM-1_-, *bla*_NDM-5_-, and *mcr-1*-positive *E. coli* strains were sequenced. The *E. coli* from different patients was assigned to ST48, ST117, ST616, ST410, ST711, and ST2003 according to the MLST scheme. The strains EC21Z-078, EC21Z-083, EC21Z-097, and EC21Z-101 isolated from the same patient belonged to ST410, and the strains EC21Z-144 and EC21Z-151 isolated from another patient belonged to ST2003. It should be noted that strains isolated from different patients presented different STs (Figure 3).

**Figure 4:**
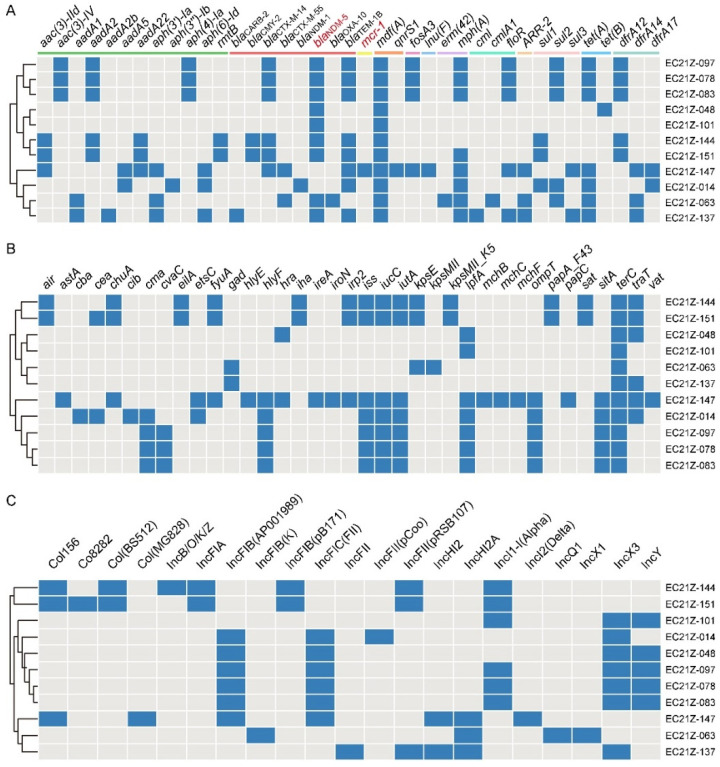
The diagram of ARGs, virulence genes, and replicon types of the 11 strains. (**A**) The blue boxes indicate the positive ARGs. (**B**) The virulence genes in *E. coli* strains. (**C**) The various plasmid replicons in *E. coli* strains.

These strains carried various ARGs encoding resistance to aminoglycoside, β-lactam, colistin, phenicol, tetracycline, sulphonamides, trimethoprim, quinolones, and others (Figure 4A). The common ARGs were *mdf*(A) (100%), *bla*_TEM-1B_(81.82%), *mph*(A) (72.72%), *tet*(A) (63.64%), and *bla*_CTX-M-14_ (54.55%), besides *bla*_NDM-5_.

There are 37 virulence genes identified from the 11 strains, with the *terC* gene detected in all strains. The virulence genes in strains from different patients are less similar (Figure 4B). The *astA* gene encodes the enteroaggregative *E. coli* (EAEC) heat-stable enterotoxin 1 (EAST1) that causes diarrheal disease [30,31]. One EAEC EC21Z-147 carrying *mcr-1* was identified from the one-day-old child. The serotype analysis showed the strain EC21Z-147 belongs to O128: H4.

Consistent with the result of S1-PFGE, several replicon types were identified in each *E. coli* strain, including B/O/K/Z, FIA, FIB, FIC, FII, HI2, HI2A, I1-I, I2, Q1, X1, X3, Y, Co8282, Col156, Col(BS512), and Col(MG828). These strains contained at least three replicons (EC21Z-101) and at most seven replicons (EC21Z-144, EC21Z-147, and EC21Z-151) (Figure 4C). The *bla*_NDM-1_ and *mcr-1* genes were located in the IncX3 (45.7 kb) and IncI2 (62.3 kb) plasmids, respectively. The plasmid of IncX3 (~46 kb) was the primary vector for the *bla*_NDM-5_ gene. The IncHI2A (109.4 kb) and IncI1-1 (~120 kb) plasmids carrying the *bla*_NDM-5_ genes were also discovered (Figure 3).

### 3.5. Plasmid Sequence and Comparative Analysis

**Figure 5:**
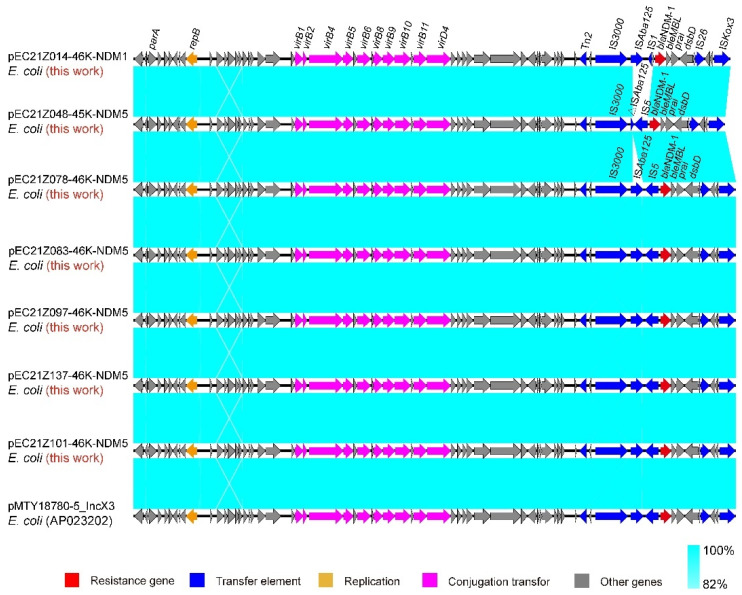
Sequence comparison of the IncX3 plasmids carrying *bla*_NDM-1_ and *bla*_NDM-5_ genes with the common plasmid backbone (pMTY18780-5_IncX3) in GenBank. Sequence similarity is shown in light blue. Arrows and triangles represent genes of different functional categories (red: resistance genes; dark blue: transfer elements; orange: replication; pink: conjugation transfer, grey: other genes).

**Figure 6:**
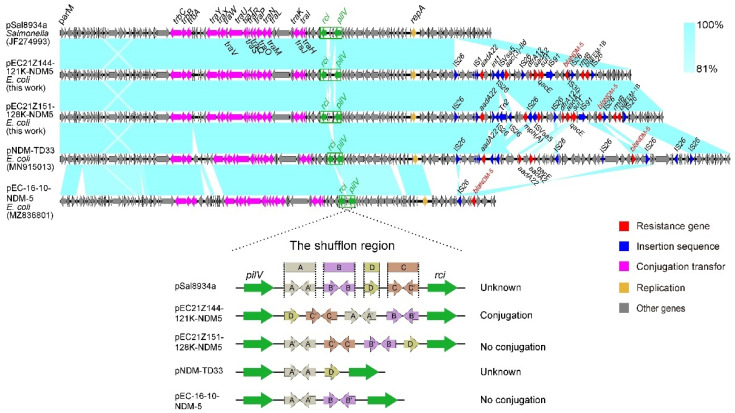
Sequence comparison of the IncHI2A plasmids carrying *bla*_NDM-5_ genes from the strains EC21Z-144 and EC21Z-151. The original plasmid (pSal8934) is compared with the pEC21Z144-121K-NDM5 and pEC21Z151-128K-NDM5. The shufflon regions are marked by a green box, and the box marking A, B, C and D referred to the four segments of shufflon. The arrows and triangles represent genes from different functional categories (red: ARGs, dark blue: insertion sequence, orange: replication, pink: conjugation transfer, green: shufflon region, grey: other genes).

**Figure 7:**
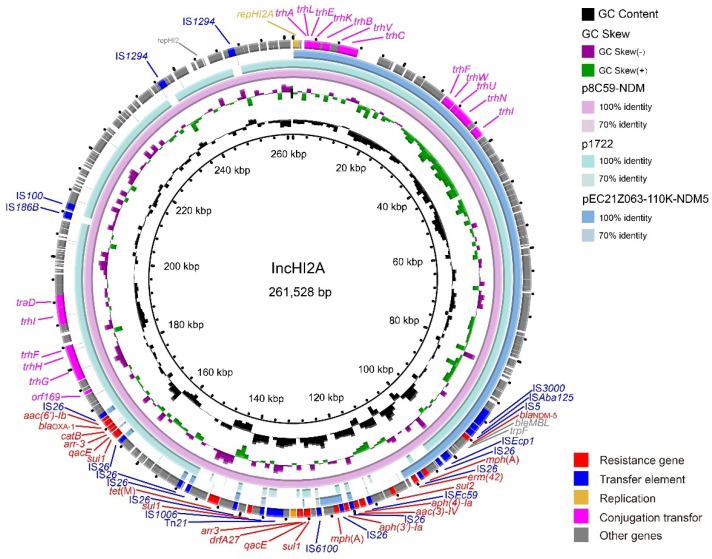
Genetic characteristics of the *bla*_NDM-5_-positive plasmid pEC21Z063-110K-NDM5 identified in this study compared with similar plasmids from pathogenic bacteria in humans or animals. The arrows and triangles represent genes from different functional categories (red: ARGs, dark blue: insertion sequence, orange: replication, pink: conjugation transfer, grey: other genes).

**Figure 8:**
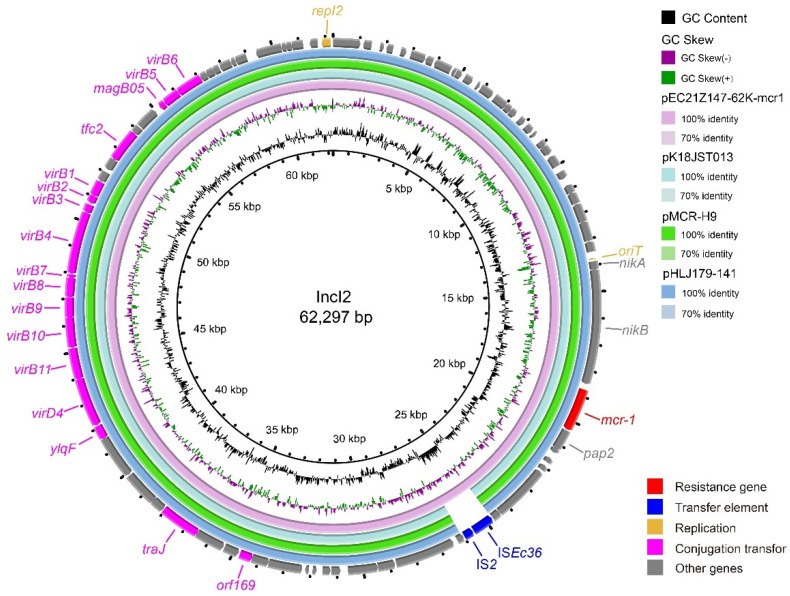
Genetic characteristics of the *mcr-1*-positive plasmid (pEC21Z147-62K-mcr1) identified in this study compared with similar plasmids from pathogenic bacteria in humans or animals. The arrows and triangles represent genes from different functional categories (red: ARGs, dark blue: insertion sequence, orange: replication, pink: conjugation transfer, grey: other genes).

The IncX3 plasmids (pEC21Z014-46K-NDM1, pEC21Z048-45K-NDM5, pEC21Z078-46K-NDM5, pEC21Z083-46K-NDM5, pEC21Z097-46K-NDM5, pEC21Z101-46K-NDM5, and pEC21Z137-46K-NDM5) carrying the *bla*_NDM-1_ or *bla*_NDM-5_ genes showed a similar sequence. All of them contained the essential elements for the conjugational system, such as the origin of transfer, relaxases, type IV coupling proteins (T4CPs), and type IV secretion system (T4SS). They were similar to the *bla*_NDM-5_-positive IncX3 plasmid (pMTY18780-5_IncX3, AP023202) in the previous study (Figure 5) [32]. The IncI1-I plasmids pEC21Z144-121K-NDM5 (120.9 kb) and pEC21Z151-128K-NDM5 (127.8 kb) carrying *bla*_NDM-5_ were isolated from one patient. The pEC21Z151-128K-NDM5 obtained an insert fragment compared with pEC21Z144-121K-NDM5 that contained macrolide resistance genes (*mph*(A) and *mrx*(A)) and the transcriptional regulator (*tet*R/*acrR*). There are two IS*26* flanked on both sides of the insert fragment that indicated the IS*26* played an important role in transmission. Furthermore, the shufflon regions of the IncI1-1 plasmid exhibited differences between pEC21Z144-121K-NDM5 and pEC21Z151-128K-NDM5 that may have affected the ability of conjugation (Figure 6). An IncI1-1 plasmid (pSal8934, JF274993) from *Salmonella enterica* was found in GenBank and has a similar genetic backbone but without the ARG (Figure 6). The pEC21Z063-110K-NDM5 carrying the *bla*_NDM-5_ gene belonged to IncHI2A, which lacked the type IV coupling protein. The similar genetic backbone plasmid p8C59-NDM (MT407547) contained a complete conjugation element (Figure 7). The pEC21Z147-62K-mcr1 carrying *mcr-1* gene belonged to IncI2, which has a typical genetic backbone and contains the essential elements for the conjugational system (Figure 8). Similar plasmids were identified in *Salmonella* (CP065423) and *E. coli* (MN232210, CP029184) from animals and humans.

The *bla*_NDM-5_ gene on the IncX3 and IncHI2A plasmids has a conservative genome context IS*3000*-IS*Aba125*-IS*5*-*bla*_NDM-5_-*ble*-*prai*-*dsbD*. The *bla*_NDM-1_ gene on the IncX3 plasmid has a similar context to IS*3000*-IS*Aba125*-IS*1*-*bla*_NDM-1_-*ble*-*prai*-*dsbD*. However, the *bla*_NDM-5_ gene was located on the IncI1-1 plasmid as IS*30*-*bla*_NDM-5_-*ble*-*prai*-*dsbD*-IS*CR1*. The *mcr-1* was located on IncI2 as a common genome context (*nikA*-*nikB*-*mcr-1*-*pap2*).

## 4. Discussion

Hospital and community settings are important reservoirs for the spread of pathogens and ARGs, such as ESBL, *bla*_NDM_, *bla*_KPC_, *mcr*, etc. Here, we investigated the prevalence of last-resort antimicrobial-resistant *E. coli* strains from inpatients in a hospital. Ten (6.37%) carbapenem-resistant-, one (0.64%) colistin-resistant-, and zero (0%) tigecycline resistant-strains were identified. All of them were found in inpatients instead of outpatients, indicating that the ARGs were spread in the hospital environment. Additionally, we observed that inpatients carry significantly more *E. coli* against last-resort antibiotics than outpatients.

The prevalence of *bla*_NDM_ (6.37%, 10/157) was higher than the global prevalence in 55 countries (0.28%, 290/103,960) [15]. It is also higher than the prevalence of CREC (2.38%, 92/3895) in healthy people in healthcare centers located in 19 provinces across China [16]. The *bla*_NDM_ gene was also detected from the specimens of various environments, vegetables (0.28%), companion animals (2.3%), and livestock and poultry (0.88%), the amounts of which were significantly lower than that in patients [33,34,35]. However, it has a similar prevalence in India (6.22%) [15]. Unlike *bla*_NDM_, the prevalence of the *mcr* and *tet*(X) genes in patients was significantly lower than that in animals, especially for the *tet*(X) gene. As expected, except the *tet*(X) gene identified in this study, these genes were more widespread in freshwater fishes (24.7%), chickens (23.6%), cattle (19.3%), and pigs (8.95%) than in patients (0.3%) [36,37]. Only one *mcr-1*-positive *E. coli* strain (0.64%) was identified in patients, similar to the positive rates in patients (0.62%, 36/5828) from several provinces of China [38]. This rate is significantly lower than those in livestock and poultry (14.81%, 270/1823), and environmental strains (5.43%, 5/92) [35,38]. It is important to note that this *mcr-1*-positive *E. coli* was isolated from the lungs in a one-day-old child. This child is the youngest reported case of *mcr-1*-positive *E. coli* infection.

The virulence gene co-located with ARGs in Enterobacteriaceae has received extensive attention in recent years. The virulence determinants were identified in various bacteria harboring *mcr*, *bla*_NDM_, or other genes from foods, animals, humans, and environments and caused a serious threat to public security [39,40,41]. The EAEC harboring heat-stable enterotoxin gene *astA* has been discovered in *E. coli* serotype O7: H4, O untypeable: H10, O4: H34, etc. The novel serotype O128: H4 *E. coli* harboring *astA* was identified in the *mcr-1*-positive *E. coli* EC21Z-14 belonging to ST117. The *E.coli* ST117 was an emerging foodborne zoonotic pathogen that has been isolated from the poultry, meat, human, and sea ecosystem [42,43,44,45]. A recent study showed that the ST117 strains isolated from different hosts had higher genetic similarity, suggesting that ST117 could transfer among different hosts [46]. In this study, the EC21Z-14 isolated from the one-day-old child was associated with the pathogenic *E. coli* from avian animals, reminding us that MDR pathogens from animals could spread in human and cause disease.

The *bla*_NDM-1_ and *bla*_NDM-5_ genes were first identified in *Klebsiella pneumoniae* in India in 2009 and in *E. coli* in the United Kingdom in 2011, respectively [1,47]. A higher prevalence of the *bla*_NDM-1_ gene (69%) than the *bla*_NDM-5_ gene (19%) was primarily reported in clinical settings [16]. However, the opposite was observed for the *bla*_NDM-5_ gene, which was dominant in CREC in China after 2016 [16]. It was observed in this study that 90% of CRECs bore the *bla*_NDM-5_ gene instead of the *bla*_NDM-1_ gene. The different hydrolysis activities may cause this to carbapenems [48]. The *bla*_NDM-5_ gene gradually replaced *bla*_NDM-1_ in China, and this phenomenon may have spread to other countries.

*bla*_NDM_-positive *E. coli* strains belonging to various STs have been reported. The most common CRECs worldwide were ST167, ST410, and ST617, and ST131 was the major CREC in China [15,48,49]. No obvious epidemic clones of NDM-positive *E. coli* were found, and only three ST410 strains were identified from one patient in this study. The strains isolated from different patients belonged to different STs. Furthermore, the mobile plasmid IncX3 is the primary replicon type for carrying various *bla*_NDM_ (NDM-1, NDM-4, NDM-5, NDM-6, NDM-7, etc.) and was detected in several strains in this study [15]. IncX3 is dominant in *bla*_NDM_-positive plasmids and promote horizontal *bla*_NDM_ gene transfer. It is pointed out that the IncX3 carrying *bla*_NDM-1_ unsuccessfully transferred to the recipient by conjugation, and no obvious mechanic was found for this abnormality. This may be a reason for the lower prevalence of the *bla*_NDM-1_ gene. IncHI2A and IncI1-1 were discovered as relatively uncommon replicon types for carrying *bla*_NDM-5_ [50]. Overall, the *bla*_NDM-5_ gene is the primarily genetic determinant for CREC, mainly spread by the IncX3 plasmids.

Multiple samples collected from two lymphoma patients at several points in time were further analyzed. The EC21Z-101 strains lost a plasmid (IncFIB-FIC) carrying several ARGs and virulence genes compared with the EC21Z-078, EC21Z-083, and EC21Z-097 strains from one patient that became sensitive to the antimicrobials. The mechanism behind the missing highly stable multidrug-resistant IncFIB plasmid is worth further studying [51]. On the other hand, the conjugation ability showed significant differences between two IncI1-1 plasmids (pEC21Z144-121K-NDM5 and pEC21Z151-128K-NDM5) carrying *bla*_NDM-5_ from one patient. Different shufflon regions were discovered between shufflon-specific DNA recombinase and PilV. It is widely known that shufflon is a multiple-inversion system that is closely related to the efficiency of the conjugation [52]. Shufflon comprises four segments (A, B, C, and D) which may be rearranged and inverted to influence the conjugation during liquid mating [53]. The rearrangement of shufflon usually reduces the transfer frequency instead of the loss [54]. The novel rearrangement in pEC21Z151-128K-NDM5 may provide a strategy to decrease plasmid dissemination, which is worth further investigating.

## 5. Conclusions

In summary, our data support that the hospital setting is a vital vehicle for spreading and disseminating last-resort AMR pathogens. Moreover, the acquisition of *bla*_NDM_ and *mcr-1* genes encoding resistance to carbapenem and colistin was mediated by horizontal transfer via plasmids. IncX3 carrying *bla*_NDM-5_ is the leading carrier of resistance in CREC. The ST117 *E. coli* is an emerging zoonotic pathogen carrying the *mcr-1* gene transferred between humans and animals. Therefore, the horizontal transfer of ARGs among inpatients indicated the importance of preventing plasmids in clinical pathogens. The continuous surveillance of carbapenem and colistin resistance at the genome level in sentinel hospitals is highly prioritized to limit the transmission of last-resort pathogens.

## Figures and Tables

**Table 1:** Source information of 11 *E. coli* isolates with meropenem- or colistin-resistant phenotype.

Patient	Sex	Age	Clinical Diagnosis	Department	Isolate	Isolation Source	Collection Date	AMR Phenotype
01235361	male	65 years	choledocholithiasis	inpatient	EC21Z-014	lung	2021/1/25	meropenem
01259916	male	73 years	cholangiolithiasis with cholangitis	inpatient	EC21Z-048	stool	2021/2/13	meropenem
01248262	female	58 years	multiple myeloma	inpatient	EC21Z-063	stool	2021/3/5	meropenem
01262726	female	61 years	diffuse large B-cell lymphoma	inpatient	EC21Z-078	stool	2021/3/29	meropenem
					EC21Z-083	lung	2021/3/29	meropenem
					EC21Z-097	stool	2021/4/6	meropenem
					EC21Z-101	lung	2021/4/13	meropenem
01275038	male	52 years	obstructive jaundice	inpatient	EC21Z-137	stool	2021/5/31	meropenem
01273986	female	49 years	non-hodgkin lymphoma	inpatient	EC21Z-144	stool	2021/6/8	meropenem
					EC21Z-151	stool	2021/6/15	meropenem
01276786	female	1 day	health	inpatient	EC21Z-147	lung	2021/6/8	colistin

**Table 2:** Antimicrobial susceptibility test of 11 *E. coli* isolates against 14 antimicrobials.

Strains	AMP	A/C	GEM	SPT	TET	FFC	SF	SXT	CEF	CAZ	ENR	OFL	MEM	COL	Antimicrobial Resistance Patterns
EC21Z-014	R	R	S	R	R	I	R	R	R	R	I	S	R	S	AMP-A/C-SPT-TET-SF-SXT-CEF-CAZ-MEM
EC21Z-048	R	R	S	S	R	I	R	S	R	R	S	S	R	S	AMP-A/C-TET-SF-CEF-CAZ-MEM
EC21Z-063	R	R	S	R	R	R	R	R	R	R	I	S	R	S	AMP-A/C-SPT-TET-FFC-SF-SXT-CEF-CAZ-MEM
EC21Z-078	R	R	I	R	R	R	R	R	R	R	R	R	R	S	AMP-A/C-SPT-TET-FFC-SF-SXT-CEF-CAZ-ENR-OFL-MEM
EC21Z-083	R	R	I	R	R	R	R	R	R	R	R	R	R	S	AMP-A/C-SPT-TET-FFC-SF-SXT-CEF-CAZ-ENR-OFL-MEM
EC21Z-097	R	R	I	R	R	R	R	R	R	R	R	R	R	S	AMP-A/C-SPT-TET-FFC-SF-SXT-CEF-CAZ-ENR-OFL-MEM
EC21Z-101	R	R	S	S	I	R	R	S	R	R	R	R	R	S	AMP-A/C-FFC-SF--CEF-CAZ-ENR-OFL-MEM
EC21Z-137	R	R	S	R	R	R	R	R	R	R	R	R	R	S	AMP-A/C-SPT-TET-FFC-SF-SXT-CEF-CAZ-ENR-OFL-MEM
EC21Z-144	R	R	R	R	S	R	R	R	R	R	R	R	R	S	AMP-A/C-GEM-SPT-FFC-SF-SXT-CEF-CAZ-ENR-OFL-MEM
EC21Z-151	R	R	R	R	S	R	R	R	R	R	R	R	R	S	AMP-A/C-GEM-SPT-FFC-SF-SXT-CEF-CAZ-ENR-OFL-MEM
EC21Z-147	R	S	R	R	R	R	R	R	R	I	R	R	S	R	AMP-GEM-SPT-TET-FFC-SF-SXT-CEF-ENR-OFL-COL
Rate (%)	100	90.9	27.2	81.8	72.7	81.8	100	81.8	100	90.9	72.7	72.7	90.9	9.1	-

## Data Availability

The nucleotide sequences presented in this study were deposited at Project accession number PRJNA856840.
